# ASIC1a Promotes Acid-Induced Autophagy in Rat Articular Chondrocytes through the AMPK/FoxO3a Pathway

**DOI:** 10.3390/ijms18102125

**Published:** 2017-10-11

**Authors:** Beibei Dai, Fei Zhu, Yong Chen, Renpeng Zhou, Zhisen Wang, Yaya Xie, Xiaoshan Wu, Shengqin Zu, Ge Li, Jinfang Ge, Feihu Chen

**Affiliations:** Anhui Key Laboratory of Bioactivity of Natural Products, School of Pharmacy, Anhui Medical University, Hefei 230032, China; dbbahmu189@163.com (B.D.); zf073326@163.com (F.Z.); cfyahmu@163.com (Y.C.); zrpgujiu@163.com (R.Z.); zswahmu@163.com (Z.W.); xyyahmu517@163.com (Y.X.); wxsahmu@163.com (X.W.); zushengqin7@163.com (S.Z.); ivory214@163.com (G.L.); gejinfang@ahmu.edu.cn (J.G.)

**Keywords:** acid, ASIC1a, Ca^2+^, AMPK, FoxO3a, autophagy, rat articular chondrocytes

## Abstract

Acid-sensing ion channel 1a (ASIC1a) is a member of the extracellular H^+^-activated cation channels family. Our previous studies suggested that ASIC1a contributed to acid-induced rat articular chondrocytes autophagy. However, its potential mechanisms remain unclear. The present study demonstrated the effect of ASIC1a on rat articular chondrocytes autophagy and explored the underlying molecular mechanisms. The results demonstrated that ASIC1a contributed to acid-induced autophagy in rat articular chondrocytes, and which was associated with an increase in (Ca^2+^)_i_, as indicated that acid-induced increases in mRNA and protein expression of LC3B-II and other autophagy-related markers were inhibited by ASIC1a-specific blocker, PcTx1 and calcium chelating agent, BAPTA-AM. Furthermore, the results showed that extracellular acid increased level of Forkhead box O (FoxO) 3a, but was reversed by inhibition of ASIC1a and Ca^2+^ influx. Moreover, gene ablation of FoxO3a prevented acid-induced increases in mRNA and protein expression of LC3B-II, Beclin1 and the formation of autophagosome. Finally, it also showed that ASIC1a activated adenine nucleotide (AMP)-activated protein kinase (AMPK). In addition, suppression of AMPK by Compound C and its small interfering RNA (siRNA) prevented acid-induced upregulation of total and nuclear FoxO3a and increases in mRNA and protein expression of LC3B-II, Beclin1, and ATG5. Taken together, these findings suggested that AMPK/FoxO3a axis plays an important role in ASIC1a-mediated autophagy in rat articular chondrocytes, which may provide novel mechanistic insight into ASIC1a effects on autophagy.

## 1. Introduction

Rheumatoid arthritis (RA) is an autoimmune disorder that affects 1% of the population in the world and characterized by chronic, multi arthritis synovial inflammation, finally leading to extra articular lesion [1]. Progressive destruction of articular cartilage, a common feature of different forms of joint diseases including RA, is a severe clinical problem [2].

Acid-sensing ion channels (ASICs), also known as voltage-independent cationic channels that belong to the epithelial sodium channel/degenerin family, can be transiently activated by extracellular acidification [3]. At pathological states, a decrease in extracellular pH activates ASICs, and then leads to the extracellular Na^+^, Ca^2+^ influx, which finally regulates cell behaviors such as apoptosis, differentiation, and autophagy [4,5,6,7,8]. However, almost all types of pathological conditions such as inflammation, ischemia, and hypoxia, variations in pH can be detected [9]. Moreover, tissue acidification is also another important pathological character in patients with RA [10] and much attention has been paid to how the sensors of articular chondrocytes adjust to these pH changes in RA. Our previous studies indicated that the activation of acid-sensing ion channel 1a (ASIC1a) contributed to the acid-induced articular chondrocytes damage which was associated with an increase in (Ca^2+^)_i_ and blockade of ASIC1a protected articular cartilage from the destruction [11,12,13,14]. However, the special mechanisms of the injury still remain unclear.

Macroautophagy (hereafter referred to as autophagy) is a complete intracellular degradation system that mainly promotes the degradation of long-lived protein to provide nutrients for survival at starvation [15,16]. Accumulating evidence indicates that autophagy can be also responsible for the degradation of damaged or excess organelles, including mitochondria and endoplasmic reticulum [17,18,19]. However, excessive activation of autophagy can lead to programmed cell death, called type II cell death [20]. It is reported that the autophagy level is increased in the synovial tissues of patients with active RA and is associated with disease severity, and, moreover, accumulating evidence showed a crucial role of autophagy in the regulation of RA [21]. ASIC1a is unique due to its highly permeable to Ca^2+^, and it is now established that intracellular Ca^2+^ is one of the regulators of autophagy [22,23]. Our previous studies have demonstrated that activation of ASIC1a by acid contributed to articular chondrocytes autophagy via extracellular Ca^2+^ influx. However, the underlying mechanisms have not yet been fully illustrated.

FoxO3a is a member of the FoxO (forkhead box O) family of transcription factors, which has been confirmed to regulate autophagy by transcriptionally upregulating autophagy-related genes (ATG) or autophagy regulatory genes [24,25]. Transcriptional activity of FoxO3a is regulated by its subcellular localization and DNA binding property, as well as by protein expression. Moreover, recent studies have found that FoxO3a is a direct downstream target of AMP-activated protein kinase (AMPK) [26] and activation of AMPK causes the nuclear shuttling of FoxO3a resulting in the expression of various target genes [27]. AMPK has been recently suggested to regulate cellular energy homeostasis through the autophagic recycling of intracellular components and play a key role in autophagy [28,29]. Furthermore, it is also reported that AMPK activation stimulated autophagy through its effects on FoxO3a for its intracellular localization and transcriptional function in skeletal muscle cells [30] and AMPK/FoxO3a axis played a cardinal role in autophagy [30,31,32]. In addition, as it reported, increasing in phosphorylation level of the AMPKα subunit at the activating threonine residue (i.e., Thr172) leads to the activation of AMPK from quiescent state to fully active state capable of phosphorylating substrates [33]. Although this increase in AMPK phosphorylation is originally considered to occur only via an AMP-dependent manner, it has been shown that the regulation of AMPK is more complex and can be also activated by some upstream kinases such as Ca^2+^/calmodulin-dependent protein kinase kinase (CaMKK) [34]. Thus, we investigated whether the AMPK/FoxO3a pathway was involved in ASIC1a-mediated autophagy in rat articular chondrocytes.

In the present study, we examined the expression of ASIC1a in rat articular chondrocytes and demonstrated again that activation of ASIC1a contributed to articular chondrocytes autophagy, which was associated with extracellular Ca^2+^ influx. We then detected the activation of AMPK/FoxO3a pathway during the regulation, suggesting that it may play a critical role in ASIC1a-mediated autophagy in rat articular chondrocytes.

## 2. Results

### 2.1. Extracellular Acidification Induces Autophagy in Rat Articular Chondrocytes

**Figure 1:**
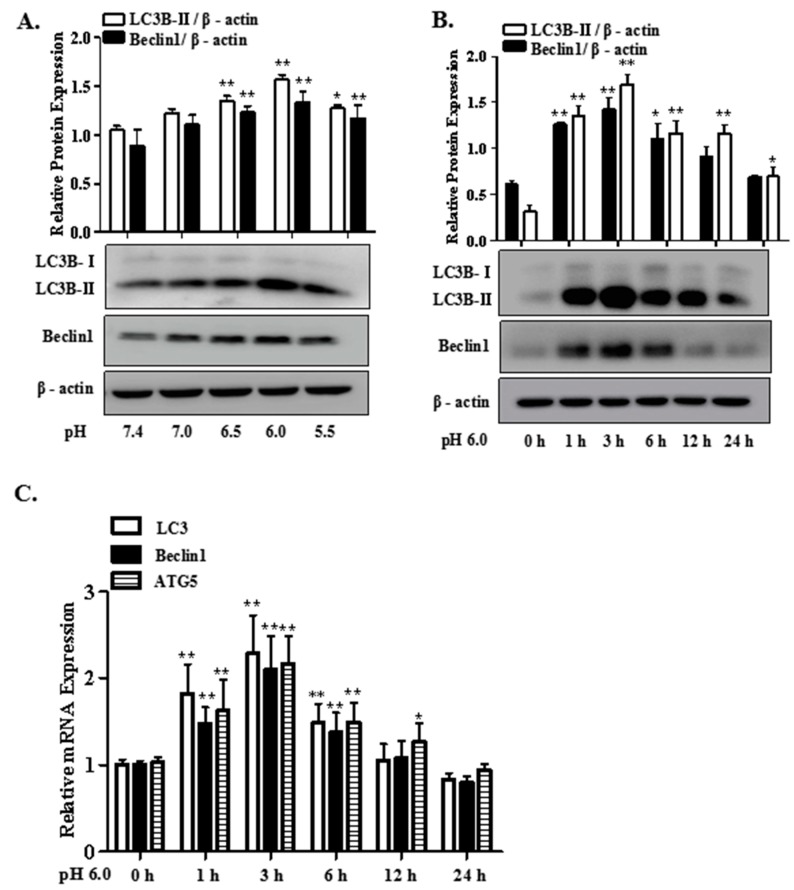
Extracellular acidification induces articular chondrocytes autophagy. (**A**) rat articular chondrocytes were treated with different concentrations of extracellular acid (pH 7.0, pH 6.5, pH 6.0, pH 5.5) for 24 h; (**B**) and (**C**) rat articular chondrocytes were treated with extracellular acid (pH 6.0) at different time points (1, 3, 6, 12 or 24 h). Then, the protein expression of LC3B-II and Beclin1 were assessed by Western blot; the mRNA expression of LC3, Beclin1 and ATG5 were analyzed by qRT-PCR. Values were presented as mean ± (Standard Deviation) SD of three independent experiments. * *p* < 0.05, ** *p* < 0.01 versus control group.

Figure 1 showed the mRNA and protein expression of the autophagy markers in articular chondrocytes treated with different pH and different time. As shown in Figure 1A,B, results of Western blot showed that the protein expression of LC3B-II and Beclin1 increased markedly after treatment with pH 6.0 for 3 h. qRT-PCR analysis also indicated that the mRNA expression of LC3, Beclin1 and ATG5 in articular chondrocytes were obviously increased at 3 h (Figure 1C). 

### 2.2. The Expression of ASIC1a in Acid-Induced Rat Articular Chondrocytes

**Figure 2:**
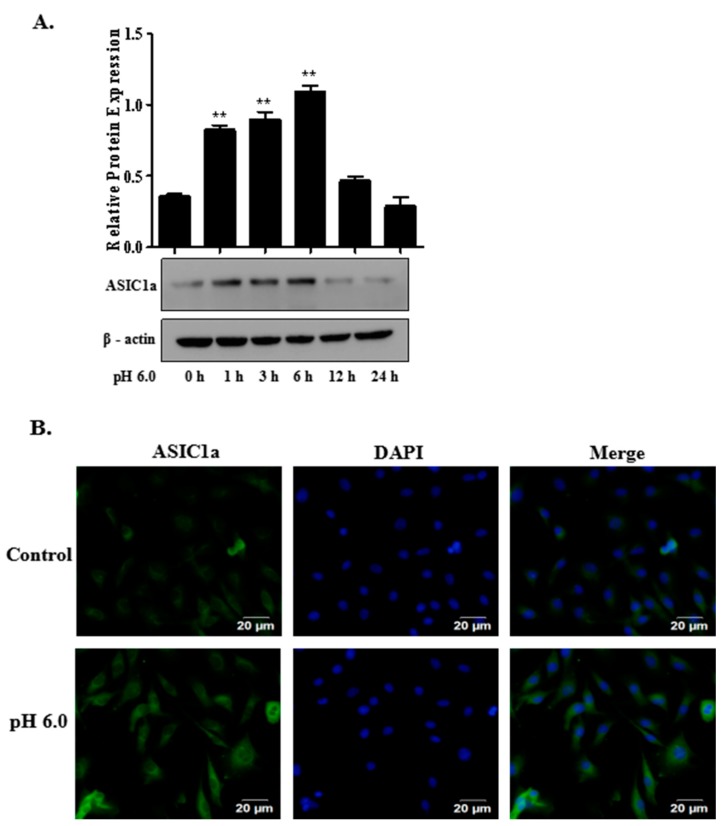
Expression of acid-sensing ion channel 1a (ASIC1a) on rat articular chondrocytes. (**A**) rat articular chondrocytes were treated with extracellular acid (pH 6.0) for different time periods. Cells were then lysed to determine the expression level of ASIC1a by Western blot analysis. Values are presented as mean ± SD of three independent experiments. ** *p* < 0.01 versus control group; (**B**) representative immunofluorescence analysis was performed on articular chondrocytes using anti-ASIC1a (green) antibody after extracellular acid (pH 6.0) treatment for 3 h. Nuclei were stained with DAPI (blue). Merge contains the combined image of ASIC1a immunostaining and DAPI staining. Scale bar = 20 μm. Determinations were done in triplicate in three independent cell cultures. DAPI: 4′,6-diamidino-2-phenylindole, a fluorescent stain that binds strongly to A-T rich regions in DNA.

To explore the expression and localization of ASIC1a, we performed Western blotting and immunofluorescent cell staining assays on rat articular chondrocytes. As shown in Figure 2A, treatment with acid increased protein expression of ASIC1a in rat articular chondrocytes. Immunofluorescent cell staining for ASIC1a was observed in the plasma membrane of articular chondrocytes and its expression was increased in acid treated cells (Figure 2B).

### 2.3. Effects of ASIC1a on Intracellular Ca^2+^ [ (Ca^2+^)_i_] in Acid-Induced Rat Articular Chondrocytes

**Figure 3:**
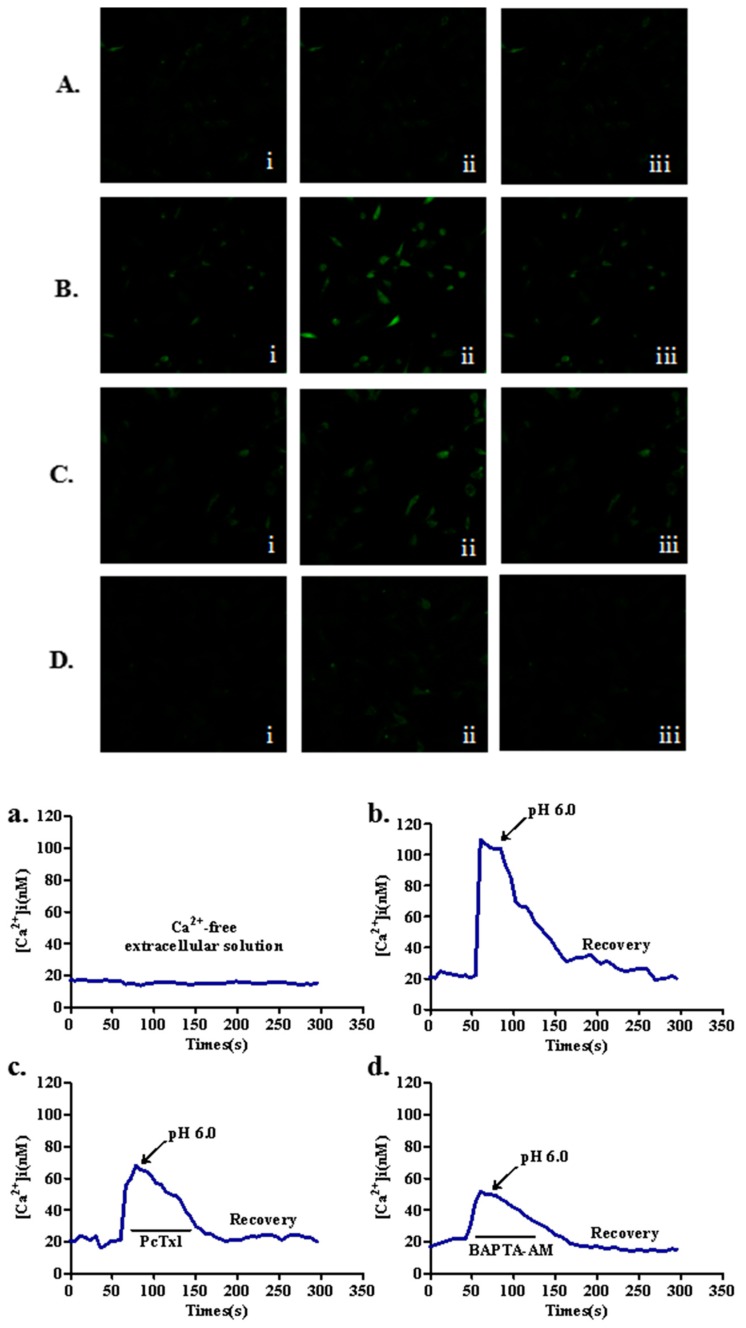
Blockade of ASIC1a with PcTx1 reduces acid-induced elevation of (Ca^2+^)_i_ level in articular chondrocytes. Cellular confocal micrographs of the same scale showing the changes in the (Ca^2+^)_i_ concentration, as visualized by Fluo-3-AM in articular chondrocytes. (**A**,**a**) acid-induced increase of (Ca^2+^)_i_ in Ca^2+^-free extracellular solution; (**B**,**b**) acid-induced increase of (Ca^2+^)_i_ in extracellular Ca^2+^ solution; (**C**,**c**) acid-induced increase of (Ca^2+^)_i_ in articular chondrocytes pretreated with ASIC1a-specific blocker, PcTx1; (**D**,**d**) acid-induced increase of (Ca^2+^)_i_ in articular chondrocytes pretreated with calcium chelating agent, BAPTA-AM. The amplitude of (Ca^2+^)_i_ intensity in articular chondrocytes induced by acid treatment was quantified as the maximal rise of (Ca^2+^)_i_ above basal levels. (**i**) before exposure to acid solution; (**ii**) increased Ca^2+^ intensity when pH was decreased to 6.0; (**iii**) after exposure to the acid solution for three minutes (*n* = 8 for each).

To investigate the role of ASIC1a in regulating the changes of (Ca^2+^)_i_, we performed laser scanning confocal microscopy in articular chondrocytes treated with acid (pH 6.0). In all experiments, blockers of glutamate receptors (10 μM, MK801), voltage-gated Ca^2+^ channels (5 μM nimodipine and 3 μM ω-conotoxin MVIIC) and 1 μM thapsigargin were added to all solutions to inhibit possible secondary activation of these channels and release of the internal Ca^2+^ stores. From the confocal micrographs and the representative traces, the results showed that acid (pH 6.0) markedly increased (Ca^2+^)_i_ in articular chondrocytes. Then, pretreatment with ASIC1a-specific blocker, PcTx1 and calcium chelating agent, BAPTA-AM could significantly reduce the concentration of intracellular calcium by acid (Figure 3).

### 2.4. Activation of ASIC1a Contributes to Acid-Induced Autophagy in Rat Articular Chondrocytes

**Figure 4:**
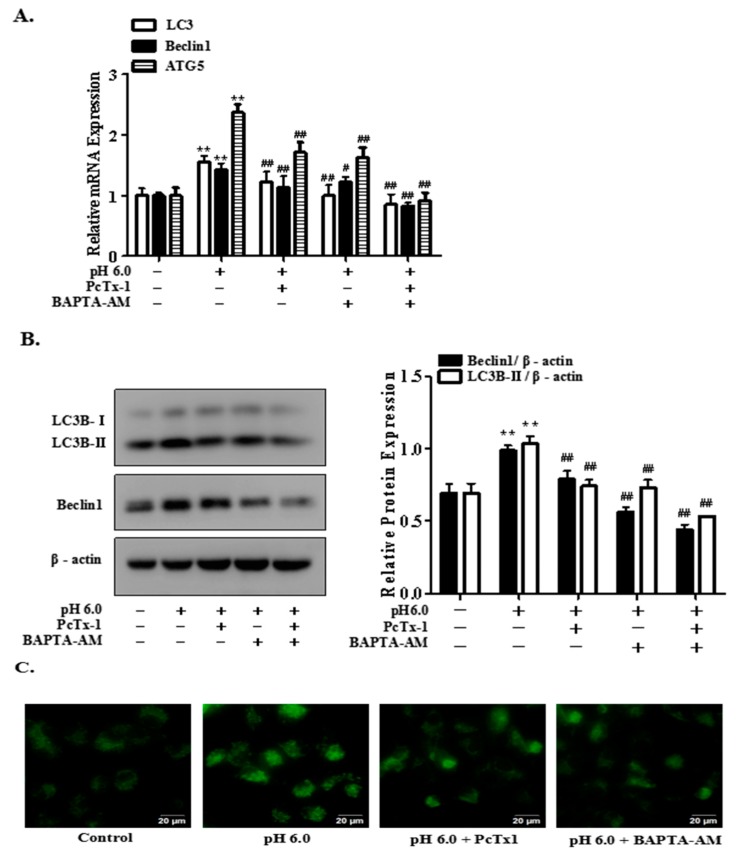
Blockade of ASIC1a and Ca^2+^ with PcTx1 and BAPTA-AM suppresses acid-induced articular chondrocytes autophagy. Rat articular chondrocytes were incubated with or without ASIC1a-specific blocker, PcTx1 (100 ng/mL) and calcium chelating agent, BAPTA-AM (10 μM) for 1 h, and then stimulated with extracellular acid (pH 6.0) for 3 h. (**A**) mRNAs were isolated from articular chondrocytes and qRT-PCR was performed; (**B**) the autophagy markers, Beclin1 and LC3B-II protein expression were determined by Western blotting; (**C**) monodansylcadaverine (MDC) staining for acidic vacuoles was analyzed by fluorescence microscopy. Scale bar = 20 μm. Values were presented as mean ± SD of three separated experiments. ** *p* < 0.01 versus control group; ^#^ *p* < 0.05, ^##^ *p* < 0.01 versus pH 6.0 group.

As shown in Figure 4, pretreatment with ASIC1a-specific blocker, PcTx1 and calcium chelating agent, BAPTA-AM significantly suppressed the mRNA and protein expression of LC3B-II, Beclin1 by acid (pH 6.0) (Figure 4A,B). In addition, to further elucidate that ASIC1a is involved in acid-induced autophagy in articular chondrocytes, we determined the autophagosome formation of the articular chondrocytes with Monodansylcadaverine (MDC) staining, which was performed to visualize the autophagosome formation with fluorescence microscopy. As shown in Figure 4C, cells treated with acid (pH 6.0) for 3 h displayed a considerable accumulation of autophagic vacuoles in the cytoplasm compared with the control group. However, autophagic vacuoles was significantly reduced in articular chondrocytes pretreated with inhibitors, compared to the cells treated with acid alone. All above results indicated that activation of ASIC1a by acid contributed to autophagy in rat articular chondrocytes, which is consistent with our previous study.

### 2.5. FoxO3a Signaling Is Involved in ASIC1a-Mediated Autophagy in Rat Articular Chondrocytes

**Figure 5:**
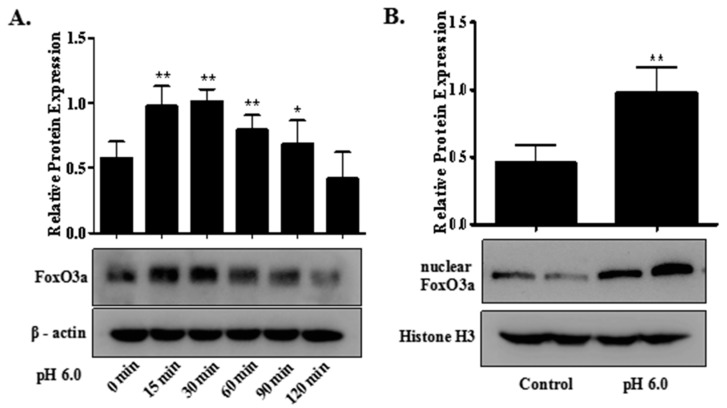

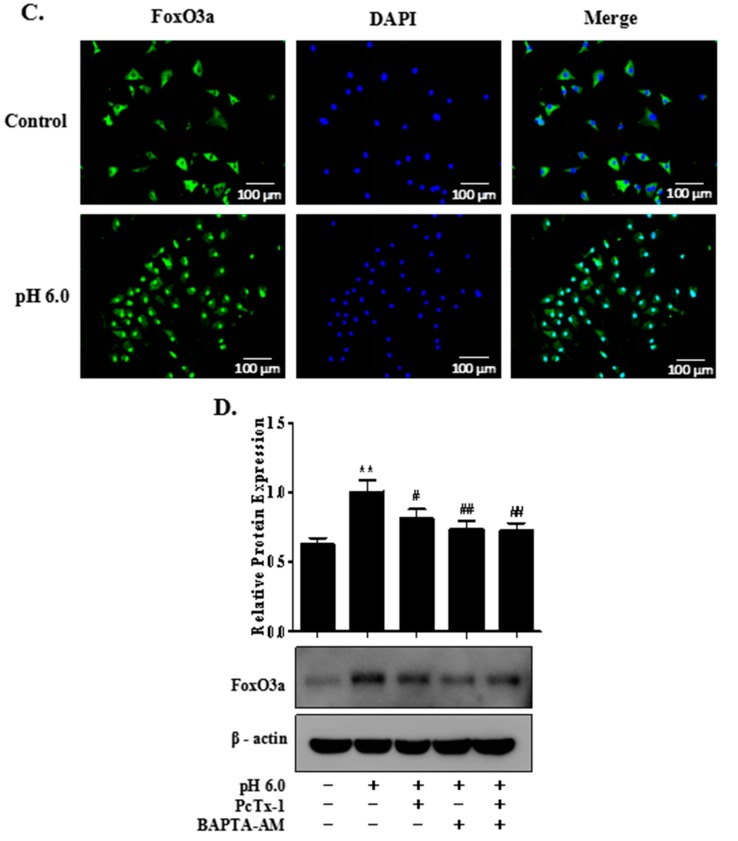

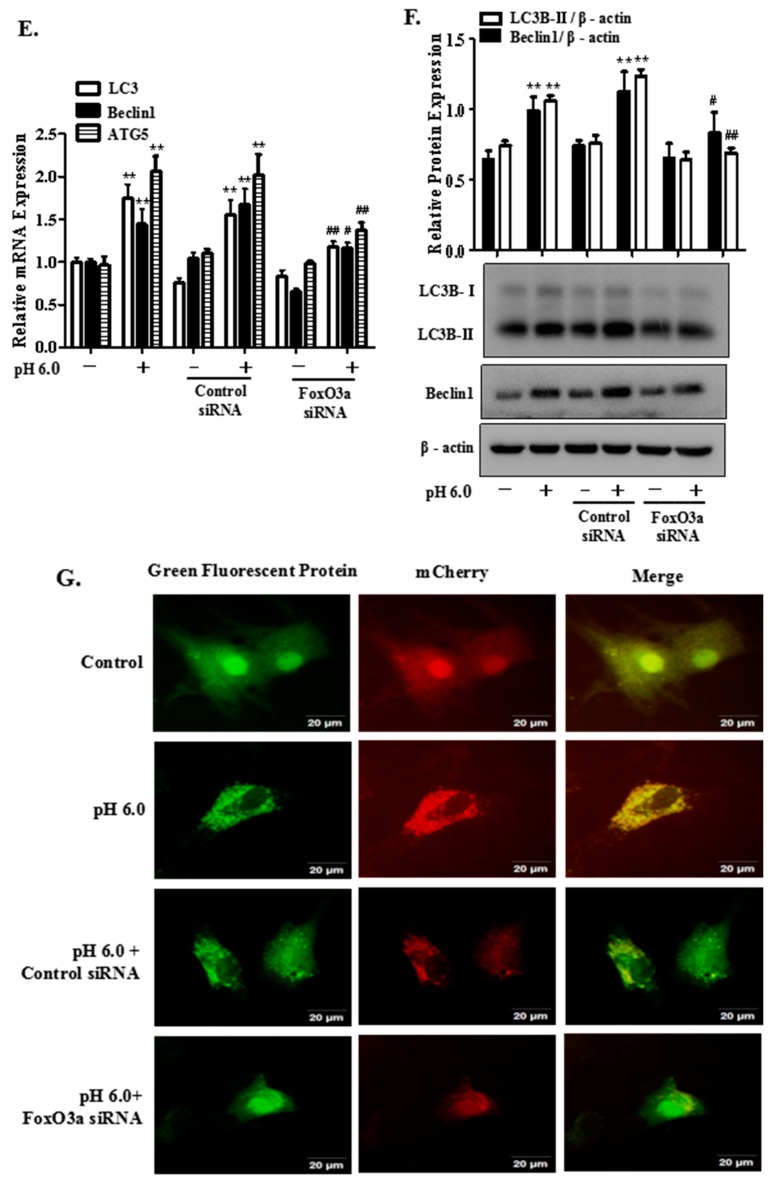

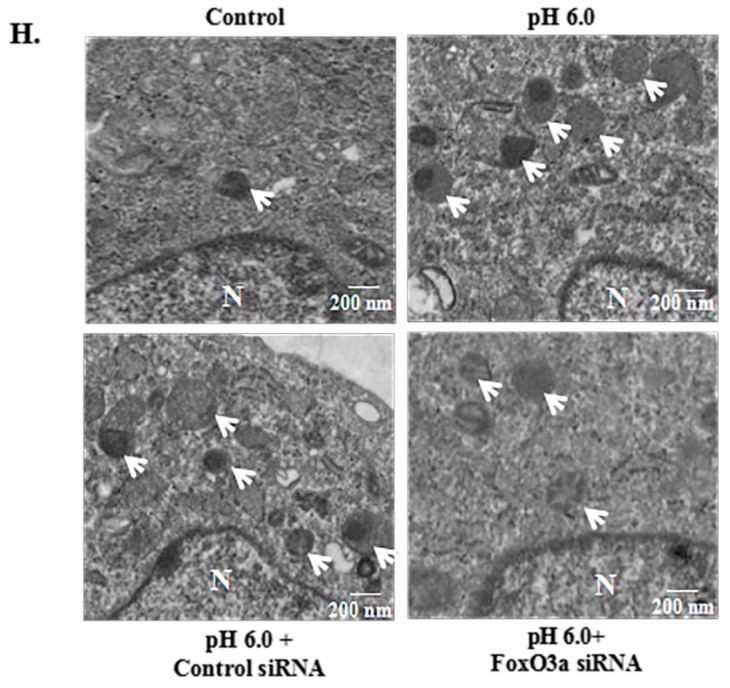
FoxO3a signaling is involved in ASIC1a-mediated articular chondrocytes autophagy. (**A**) rat articular chondrocytes were incubated with extracellular acid (pH 6.0) for the indicated time periods. The protein expression of FoxO3a was assessed by Western blot; (**B**) nuclear and cytoplasmic FoxO3a proteins were isolated as described in materials and methods and expression level of FoxO3a in nuclear fraction was determined by Western blot analysis; histone H3 was used as normalized control in nuclear protein; (**C**) representative immunofluorescence analysis was performed on articular chondrocytes using anti-FoxO3a (green) antibody after acid treatment for 30 min. Nuclei were counterstained with DAPI (blue). Merge contains the combined image of FoxO3a immunostaining and DAPI staining. Scale bar = 100 μm. DAPI: 4′,6-diamidino-2-phenylindole, a fluorescent stain that binds strongly to A-T rich regions in DNA. (**D**) pretreatment with ASIC1a-specific blocker, PcTx1 and calcium chelating agent, BAPTA-AM for 1 h, articular chondrocytes were stimulated with extracellular acid (pH 6.0) for 30 min. The FoxO3a protein expression was determined by Western blotting; (**E**,**F**) rat articular chondrocytes were transfected with siRNA targeting FoxO3a or scrambled control siRNA. After transfection, articular chondrocytes were treated with extracellular acid; (**E**) mRNAs were isolated from articular chondrocytes and qRT-PCR was performed; (**F**) the protein expression of Beclin1, LC3B-II were determined by Western blot analysis; (**G**) rat articular chondrocytes were transfected with a mCherry-EGFP-LC3B plasmid and FoxO3a-siRNA or control-siRNA and then treated with extracellular acid, the GFP-LC3 puncta (green dots) and autophagosomes (yellow dots) and autolysosomes (red dots) were analyzed by fluorescence microscopy. Scale bar = 20 μm; (**H**) rat articular chondrocytes were transfected with FoxO3a-siRNA or control-siRNA and then treated with extracellular acid. The ultrastructure of the chondrocytes was imaged using transmission electron microscopy. N: nucleus; White arrows: autophagosomes. All images were shown at 20,000× magnification. Data were presented as mean ± SD of three independent experiments. * *p* < 0.05, ** *p* < 0.01 versus control group; ^#^ *p* < 0.05, ^##^ *p* < 0.01 versus pH 6.0 group.

In the present study, to investigate whether extracellular acid (pH 6.0) activated FoxO3a and in turn increased the transcription of autophagy-related genes, some assays were performed in rat articular chondrocytes. As shown in Figure 5A, prolonged treatment with acid caused increased expression of FoxO3a and showed a peak at 30 min. More importantly, Western blot analysis for nuclear fractions and immunofluorescence showed that acid treatment increased nuclear FoxO3a level approximately twofold in rat articular chondrocytes (Figure 5B,C). However, pretreatment with ASIC1a-specific blocker, PcTx1 and calcium chelating agent, BAPTA-AM could reduce protein expression of FoxO3a in acid-treated articular chondrocytes (Figure 5D). To examine whether FoxO3a activation was responsible for the increased autophagy, we utilized the siRNA targeting FoxO3a to knockdown the acid-promoted FoxO3a. It was demonstrated that promoted mRNA and protein expression of LC3B-II, Beclin1 and ATG5 by acid (for 3 h) could be markedly reduced by the transfection with siRNA-FoxO3a (Figure 5E,F). Next, mCherry-EGFP-LC3B plasmid was used to monitor autophagic flux by fluorescence microscopic analysis. After transiently transfected rat articular chondrocytes with mCherry-EGFP-LC3B plasmid, acid treatment clearly elevated the portion of autolysosomes compared to control and knocking down of the *foxo3a* gene abolished the formation of autophagosome by acid (Figure 5G). Moreover, our evidence for the role of FoxO3a in ASIC1a-mediated autophagy was substantiated by transmission electron microscopy (TEM), the most convincing and standard method to detect autophagy [35]. As shown in the electron micrographs, the increased number of double-membrane autophagy vesicles containing subcellular materials was observed in acid-treated articular chondrocytes, but the number of autophagosomes in cells treated with siRNA-FoxO3a was diminished compared to acid treatment (Figure 5H). These findings indicated that ASIC1a contributed to activation of FoxO3a, and provided critical evidence for the role of FoxO3a signaling in ASIC1a-mediated autophagy in rat articular chondrocytes.

### 2.6. AMPK/FoxO3a Signaling Is Required for ASIC1a-Mediated Autophagy in Rat Articular Chondrocytes

**Figure 6:**
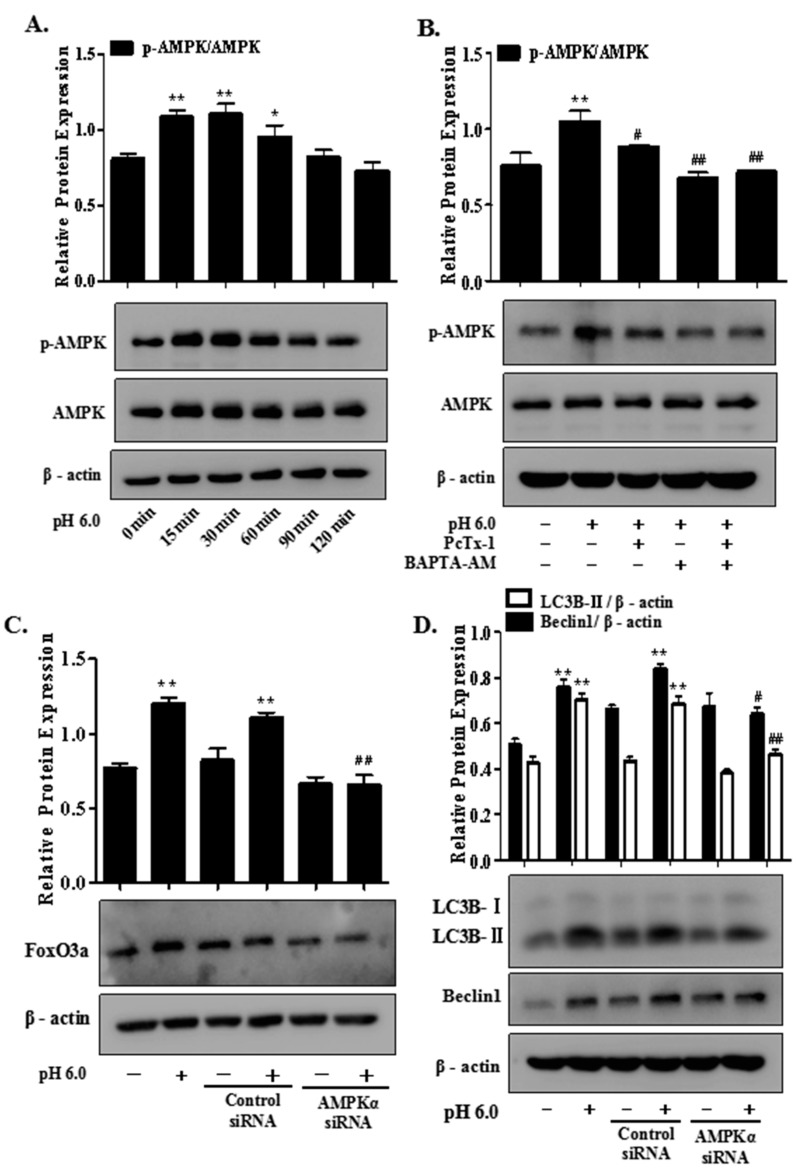

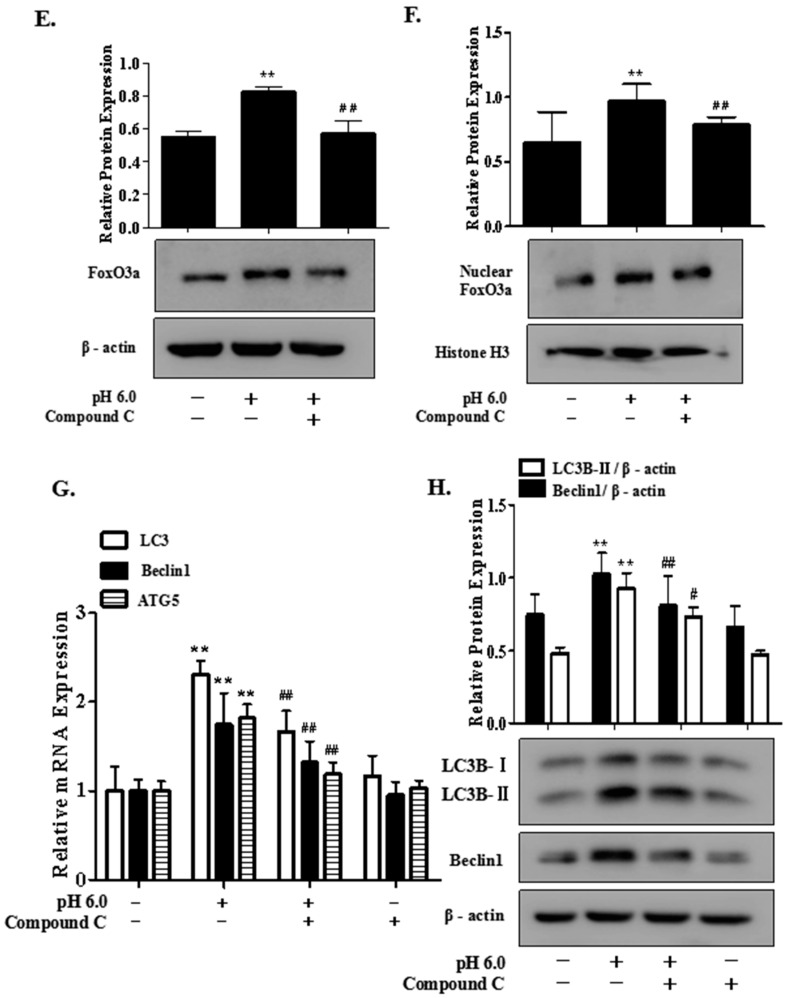

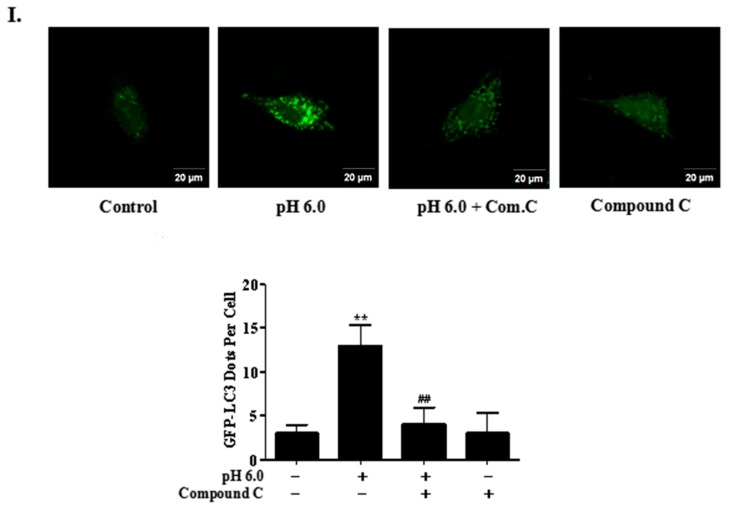
AMPK/FoxO3a signaling is involved in ASIC1a-mediated articular chondrocytes autophagy. (**A**) rat articular chondrocytes were treated with extracellular acid (pH 6.0) for the indicated time periods. Cells were then lysed to determine the expression level of phosphorylated AMPKα (Thr172) by Western blot analysis; (**B**) pretreatment with ASIC1a-specific blocker, PcTx1 and calcium chelating agent, BAPTA-AM for 1 h, articular chondrocytes were stimulated with extracellular acid (pH 6.0) for 30 min. The level of p-AMPK/AMPK was determined by Western blotting; (**C**) rat articular chondrocytes were transfected with siRNA specific for AMPKα or scrambled control and then treated with extracellular acid (pH 6.0). After 30 min incubation, cells were then lysed to determine the expression level of FoxO3a by Western blot analysis; (**D**) rat articular chondrocytes transfected with siRNA for AMPKα or scrambled control were treated with extracellular acid. The levels of Beclin1, LC3B-II protein expression were determined by Western blot followed by densitometry analysis; (**E**,**F**) rat articular chondrocytes were pretreated with Compound C (10 µM) followed by treatment with extracellular acid (pH 6.0) for 30 min. The protein expression levels of total and nuclear FoxO3a were examined by Western blotting; (**G**,**H**) rat articular chondrocytes were incubated with or without 10 μM Compound C for 1 h and then stimulated with extracellular acid (pH 6.0) for 3 h. The mRNA and protein expression of Beclin1, LC3B-II were determined by qRT-PCR and Western blotting; (**I**) articular chondrocytes were transfected with EGFP-LC3 plasmid and then treated as for (**H**) followed by examining with fluorescence microscopy. Scale bar = 20 μm. GFP-LC3 puncta were quantified for each experiment, with at least 30 cells counted in each experiment. Data were presented as mean ± SD of three independent experiments. * *p* < 0.05, ** *p* < 0.01 versus control group; ^#^ *p* < 0.05, ^##^ *p* < 0.01 versus pH 6.0 group.

In a continuing study to identify the upstream signaling molecule mediating FoxO3a activation and cell autophagy, we examined the involvement of AMPK signaling. For this, we first confirmed that treatment with acid (pH 6.0) led to a rapid phosphorylation of AMPK (Figure 6A) and pretreatment with ASIC1a-specific blocker, PcTx1 and calcium chelating agent, BAPTA-AM could decrease activation of AMPK in acid-treated articular chondrocytes (Figure 6B). Moreover, gene silencing of AMPK prevented acid-induced increased expression of FoxO3a (Figure 6C). We next investigated the functional role of AMPK signaling in ASIC1a-mediated autophagy. As shown in Figure 6D, the protein expression of LC3B-II and Beclin1 in acid-treated articular chondrocytes were significantly suppressed by transfection with siRNA targeting AMPK. Pretreatment with Compound C, a pharmacological inhibitor of AMPK, showed the similar results to those from gene silencing of AMPK that Compound C suppressed the acid-induced increased expression of total and nuclear FoxO3a (Figure 6E,F). In addition, Compound C also significantly suppressed acid-induced increases in the mRNA and protein expression of LC3B-II and Beclin1 and the number of GFP-LC3 puncta in articular chondrocytes (Figure 6G,H,I). All of these results indicated that ASIC1a contributed to extracellular acid-induced activation of AMPK/FoxO3a axis, which played an important role in the regulation of ASIC1a-mediated articular chondrocytes autophagy.

### 2.7. Proposed Model for the Role of ASIC1a in Acid-Induced Autophagy in Rat Articular Chondrocytes

**Figure 7:**
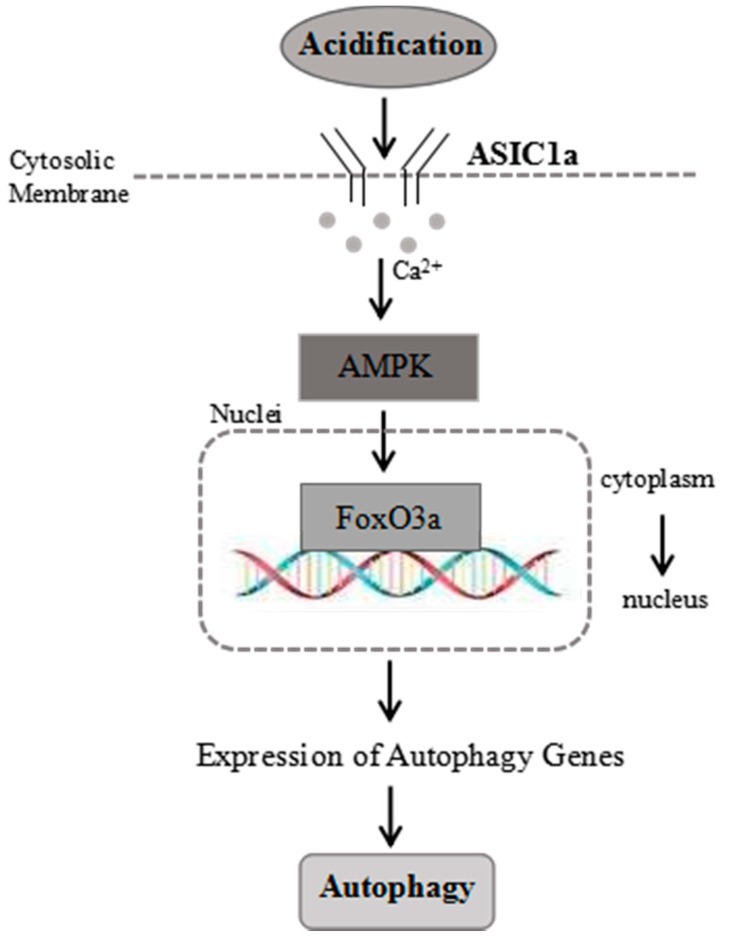
Proposed model for the role of AMPK/FoxO3a signaling in the ASIC1a-mediated autophagy in rat articular chondrocytes.

As shown in Figure 7, treatment with acid triggers activation of ASIC1a, and then leads to the extracellular Ca^2+^ influx, which, in turn, activates AMPK. Then, AMPK signaling causes activation of FoxO3a for its over-expression and translocation into the nucleus. ASIC1a promotes acid-induced autophagy and the AMPK/FoxO3a axis plays a critical role in ASIC1a-mediated autophagy in rat articular chondrocytes via induction of autophagy related genes expression, which, in turn, activates autophagy.

## 3. Discussion

In this study, we explored the role of AMPK/FoxO3a pathway in regulating ASIC1a-mediated articular chondrocyte autophagy. The results showed that ASIC1a contributed to acid-induced autophagy via increasing intracellular Ca^2+^ in rat articular chondrocytes. Acid (pH 6.0) treatment could upregulate the level of ASIC1a and blockade with PcTx1 and BAPTA-AM suppressed acid-induced autophagy in articular chondrocytes. Furthermore, the AMPK/FoxO3a axis has been proved to be involved in ASIC1a-mediated autophagy in rat articular chondrocytes. Acid (pH 6.0) treatment could activate AMPK and upregulate the levels of total and nuclear FoxO3a, which could be reversed by the blockage of ASIC1a with PcTx1. Gene silencing of AMPK and FoxO3a could reduce the mRNA and protein expression of LC3B-II and other autophagy related markers in acid-induced articular chondrocytes.

Chondrocytes play a vital effect on maintaining cartilage tissue homeostasis as a role cell type in cartilage. During diseased states including overloading, hypoxia as well as acidosis, chondrocytes are also regulated and finally lead to irreversible cartilage degeneration [36]. Although the physiopathology of RA is complex, articular chondrocytes damage is often considered as an essential triggering event for the development of RA.

Autophagy was initially identified as a cellular housekeeping pathway used to remove damaged protein and organelles, complete additional energy and resynthesize vital macromolecules during nutrient starvation [37,38]. However, autophagy not only plays cytoprotective functions, but also contributes to cell death in many types of cells. Some studies demonstrated that autophagy is increased in the chondrocytes and cartilage of osteoarthritis (OA) patients in response to catabolic and nutritional stresses, and the increased autophagy provides important protection effects from cartilage destruction and apoptosis [38]. Autophagy has emerged as a mediator in the cartilage destruction. Thus, it is very significant to explore autophagy process during articular cartilage destruction of RA.

ASICs are proton-gated cation voltage-independent cationic channels that are exquisitely susceptible to a drop reduction of extracellular pH. To date, at least seven different ASIC subunit proteins (ASIC1a, ASIC1b, ASIC1b_2_, ASIC2a, ASIC2b, ASIC3, ASIC4) encoded by four separate genes (Accn1, Accn2, Accn3 and Accn4) have been identified in mammalian [39,40]. As it reported, intracellular Ca^2+^ is a ubiquitous second messenger in signal transduction that regulates diverse physiological functions. Interestingly, activation of ASICs is involved in Na^+^ and Ca^2+^ flux [8]. Joint acidosis, a vital component of the pathogenic factors associated with RA, is characterized by an increase in the concentration of hydrogen ions in the synovial fluid. Our previous study has demonstrated that extracellular acidosis could activate ASICs, especially the Ca^2+^-permeable ASIC1a that induced (Ca^2+^)_i_ overload, which ultimately contributed to acid-induced rat articular chondrocytes injury [14]. We further found that a blockade of ASICs with amiloride could alleviate articular cartilage destruction in adjuvant arthritis rats [41]. In the present study, our results showed that ASIC1a promoted autophagy in acid-induced articular chondrocytes by increasing intracellular calcium concentrations, which was consistent with the results in our previous study. In addition, unraveling the molecular mechanisms underlying autophagy, we investigated the involvement of AMPK and FoxO3a signaling in ASIC1a-mediated autophagy in articular chondrocytes.

It has been demonstrated that the transcriptional activities of FoxOs are required for the induction of autophagy in many tissues, such as skeletal muscle [25,42], cardiomyocytes [43], kidney [44], liver [45], and cancer cells [46]. FoxO3a transcription factor has been proved to have a protective role in chondrocytes by regulation of autophagy and defending oxidative stress [47]. Furthermore, during the pathological processes of aging and OA, FoxOs were involved in the regulation of autophagy [48]. In this study, FoxO3a-mediated transcription of autophagy-related genes played a critical role in ASIC1a-mediated autophagy in rat articular chondrocytes. This notion is supported by the following evidence: (a) acid treatment increased expression level and promoted nuclear localization of FoxO3a; (b) ASIC1a regulated activation of FoxO3a; and (c) knocking down FoxO3a inhibited acid-induced expression of autophagy-related genes, resulting in reduced autophagy.

In addition to the well-known metabolic functions, recent studies have shown that AMPK is sufficient and necessary for induction of autophagy by mediating autophagosome formation and LC3 lipidation [49,50,51,52,53,54]. Two upstream kinases that promote AMPK phosphorylation are the tumor suppressor Liver Kinase B1 (LKB1) and Ca^2+^-calmodulin-activated protein kinase kinase β, CamKKβ [55,56,57,58]. Activation by CamKKβ is mediated by increases in cytosolic Ca^2+^ concentrations, thus providing another pathway to activate AMPK independent of changes in adenine nucleotide concentration. In addition, AMPK, referred as upstream kinase is also able to associate with and phosphorylate FoxOs both in nematodes and mammalian cells [59]. In the present study, we have further demonstrated the detailed downstream signaling of AMPK, including activation of nuclear FoxO3a and further modulating expression of genes involved in regulation of autophagy. Herein, we clearly showed that AMPK signaling plays a critical role in ASIC1a-mediated articular chondrocytes autophagy via activation of FoxO3a. In addition, it is recently reported that FoxO3a transcription factor activation by AMPK induced the expression of the autophagy-related proteins such as LC3B-II, Gabarapl1, and Beclin1 in primary mouse skeletal muscle myotubes and in the Tibialis anterior (TA) muscle [30], suggesting a crucial role of AMPK/FoxO3a in the regulation of autophagy, which showed similar mechanisms to those from ASIC1a-mediated induction of autophagy. Although previous studies have also indicated the induction of autophagy in acid-induced articular chondrocytes, the detailed molecular mechanisms have not been fully demonstrated. Herein, we clearly demonstrated that AMPK/FoxO3a axis is involved in ASIC1a-mediated autophagy in articular chondrocytes.

## 4. Materials and Methods

### 4.1. Cell Culture and Treatment

The primary rat articular chondrocytes were obtained and cultured as we described previously [13]. Briefly, rat articular chondrocytes were obtained from young adult male Sprague–Dawley rats weighing 160–180 g purchased from the Center for Laboratory Animal Sciences at Anhui Medical University (Hefei, China). All animal experimental procedures were in compliance with the guidelines of the Ethics Committee of Anhui Medical University. Cartilage tissues from knee joint of rats were pooled for each isolation procedure. Cartilage tissues were finely cut into small pieces (~1 mm^3^), and then digested with 0.2% type II collagenase (Sigma Chemical Co., St. Louis, MO, USA) at 37 °C for 6 h in phosphate buffered saline (PBS, Boster, Wuhan, China). After digestion, isolated chondrocytes were washed three times with PBS. The primary cells were cultured in 25 mL cell culture plates (Corning Inc., Corning, NY, USA) filled with Dulbecco’s Modified Eagle’s Medium (DMEM, Gibco, Grand Island, NY, USA) supplemented with 10% fetal bovine serum (FBS, Gibco) and antibiotics (Invitrogen Corp., Carlsbad, CA, USA) at a density of 2 × 10^4^ cells/cm^2^. The cells were cultured under sterile conditions at 37 °C in 5% CO_2_ incubator and used within the first three passages. For acidic stimulation, the extracellular pH was adjusted by the addition of an appropriate amount of HCl to achieve different pH values [6]. Inhibition experiments were carried out by pretreatment for 1 h with ASIC1a-specific blocker, PcTx1 (100 ng/mL; Abcam, Cambridge, MA, USA), calcium chelating agent, BAPTA-AM (10 uM; Sigma-Aldrich, St. Louis, MO, USA) and AMPK inhibitor, Compound C (10 uM; Selleck, Shanghai, China). The effective concentration of PcTx1, BAPTA-AM, Compound C was obtained from the literature.

### 4.2. Immunofluorescence Assay 

After treatment as indicated earlier, the articular chondrocytes were fixed with ice-acetone for 15 min, permeabilized with 0.3% Triton X-100 in PBS for 15 min, and then blocked with PBS containing 5% bovine serum albumin (BSA, Sigma-Aldrich) for 1 h. The cells were then incubated with anti-ASIC1a (1:100, Alomone Labs, Jerusalem, Israel), anti-FoxO3a (1:100; Cell Signaling Technology, Danvers, MA, USA) antibody overnight at 4 °C, followed by detection with an fluorescein isothiocyanate (FITC)-conjugated anti-rat IgG (Molecular Probes, Beijing, China) in the dark for 1 h at 37 °C. Control sections were treated in parallel with a non-immune isotype-matched IgG substituting for the primary antibody. Nuclear staining was incubated with 4′,6-diamidino-2-phenylindole, dilactate (DAPI; Invitrogen, Carlsbad, CA, USA). Cells were washed and imaged using an inverted fluorescence microscope (Olympus, Tokyo, Japan).

### 4.3. Laser Scanning Confocal Microscopy

The intracellular calcium of rat articular chondrocytes was monitored by fluorescence imaging [60]. Cells (2 × 10^5^) on coverslips were washed three times with D-Hanks’ soon and incubated with 0.02% Pluronic F-127 and 4 μM Fluo-3-AM (Biotium, Hayward, CA, USA) at 37 °C for 30 min. After incubation, the cells were washed twice with Hank’s solution to clear away extra Fluo-3-AM. To eliminate the effects of voltage-gated Ca^2+^ channels, ω-Conotoxin MVIIC (3 μM) and nimodipine (5 μM) were added to the extracellular solution. The fluorescence of intracellular Fluo-3 was measured by confocal laser scanning fluorescence microscopy (Carl-Zeiss, Jena, Germany) at a excitation wavelength of 488 nm and emission wavelength of 525 nm, respectively. Gray scale images were collected using fluorescence microscopy at different time points from 0 to 5 min and then archived as TIFF image files for later analysis. Significant images were analyzed with Leica-sp5 Leica Application Suite (LAS) AF software (Leica Microsystems Inc., Buffalo Grove, IL, USA).

### 4.4. MDC Labeling

MDC is an acid dye and a specific marker for autophagic vacuoles. MDC staining used for detecting and quantifying autophagy was measured by florescence microscopy. Briefly, after treatment as indicated earlier, chondrocytes were washed with ice-cold PBS, and then incubated with 100 μM of MDC at 37 °C for 30 min in the dark. The stained cells were washed and immediately analyzed under a florescence microscope.

### 4.5. Transmission Electron Microscopy

After treatment, cells were harvested by centrifuging at 1500 rpm for 5 min, washed with PBS and fixed for 4–6 h with pre-cold 2.5% glutaraldehyde, and then post-fixed with 1% osmium tetroxide for 1 h. Then, the samples were rinsed with grade ethanol (50%, 70%, 80%, 90%, 95%). After dehydration, the cells were exposed to Epon 812 and polymerized at 45 °C for 12 h, 65 °C for 48 h. The epoxy-embedded blocks were sectioned with an ultramicrotome (LKB-NONA, Bromma, Sweden). Thin sections were double stained with uranium acetate saturated aqueous solution and use citrate for contrast staining, and finally observed by TEM (JEM-1230, JEOL, Tokyo, Japan).

### 4.6. Small Interfering RNA (siRNA) and Plasmid Transfection

Articular chondrocytes were cultured in Opti-MEM (Gibco) medium, immediately, corresponding siRNA for target genes or scrambled control siRNA or mCherry-EGFP-LC3B or EGFP-LC3B plasmid were transfected into chondrocytes using Lipofectamine 2000 reagent (Invitrogen, Carlsbad, CA, USA). Six to eight hours later, the cells were resuspended in a complete medium for the following experiments. The FoxO3a, AMPKα specific siRNA and EGFP-LC3B plasmid was synthesized by GenePharma (Shanghai, China). pBABE-puro mCherry-EGFP-LC3B was a gift from Jayanta Debnath (Addgene plasmid # 22418). The siRNA duplexes are listed in Table 1.

### 4.7. Total RNA Extraction and Quantitative Real-Time PCR (qRT-PCR)

Total RNA was extracted from chondrocytes with TRIzol reagent (Invitrogen Corp., Carlsbad, CA, USA), and then immediately performed reverse transcription using First Strand cDNA Synthesis Kit (Thermo Fisher Scientific, Waltham, MA, USA) as the manufacturer’s protocol. qRT-PCR analysis for mRNA of LC3, Beclin1, ATG5 and β-actin were conducted by PIKO REAL RT-PCR kits (Thermo Fisher Scientific). The mRNA level of β-actin was used as the endogenous control. The primers sequences were shown in Table 2. PCR was performed at 95 °C for 10 min, followed by 40 cycles of amplification at 95 °C for 15 s, 60 °C for 30 s and 72 °C for 30 s with PIKO REAL 96 (Thermo Fisher Scientific). Relative expression levels were analyzed by 2^-ΔΔC*t*^ method. The PCR was performed in triplicate and repeated at least three times.

### 4.8. Western Blot Analysis

RIPA lysis buffer (Beyotime, Shanghai, China) was used to lyse rat articular chondrocytes. The nuclear protein and cytoplasmic fractions were extracted using a nuclear and cytoplasmic protein extraction kit (Beyotime, Shanghai, China) according to the manufacturer’s protocol. The concentrations of protein were detected using a BCA protein assay kit (Boster, Wuhan, China). Total cell lysates (30 μg proteins) were separated by SDS-PAGE (10%, 80 V for 30 min and then 120 V for 90 min) (Bio-Rad Laboratories Inc., Berkeley, CA, USA) and electrotransferred onto a polyvinylidene fluoride (PVDF) membrane (Millipore Corp., Billerica, MA, USA). Then, the PVDF membranes were blocked with 5% non-fat dry milk in Tris buffered saline Tween 20 (TBS-T) (3 h) at room temperature. After blocking, the PVDF membranes were probed with specific primary antibodies overnight at 4 °C. Guinea pig polyclonal anti-ASIC1a (Alomone Labs, Jerusalem, Israel) was applied at a dilution of 1:600, rabbit monoclonal antibodies against LC3B, Beclin1, FoxO3a, AMPKα, phospho-AMPKα (Cell Signaling Technology, Danvers, MA, USA) were used at dilution of 1:1000, mouse monoclonal anti-β-actin (Santa Cruz Biotechnology, Santa Cruz, CA, USA) was used at a dilution of 1:500. Horseradish peroxidase (HRP)-conjugated anti-rabbit and anti-mouse antibodies were correspondingly used as secondary antibody. The membrane was rinsed with TBS-T for another three times, and the chemiluminescent bands were developed using ECL-chemiluminescent kit (ECL-plus, Thermo Fisher Scientific). Autoradiographs were scanned using an Image-Pro Plus image analysis software (Media Cybernetics, Rockville, MD, USA). All of the experiments reported in this study were performed three times and the results were reproducible.

### 4.9. Statistical Analysis

The data were expressed as the mean ± SD with SPSS 17.0 software (SPSS Inc., Chicago, IL, USA). Statistical analysis was performed using the one-way ANOVA and unpaired Student’s *t*-test for the comparison among the different treatment groups. Degrees of significance were defined by *p* < 0.05. The results shown were representative of at least three separate experiments.

## 5. Conclusions

In conclusion, we have demonstrated here that activation of ASIC1a is responsible for acid-induced rat articular chondrocytes autophagy, and which is mediated by the AMPK/FoxO3a pathway. More importantly, ASIC1a-mediated increased (Ca^2+^)_i_ acts as an upstream molecule leading to the activation of AMPK and FoxO3a in articular chondrocytes treated with acid. The present study suggests that AMPK/FoxO3a axis would be a novel mechanism for the ASIC1a-mediated autophagy in articular chondrocytes and further modulation of AMPK/FoxO3a axis would be a promising therapeutic strategy in articular chondrocytes’ damage of RA development.

## Figures and Tables

**Table 1:** Sequences of small interfering RNA used in transfection.

Target Gene	Forward Primer	Reverse Primer
FoxO3a	5′-CACCAUGAAUCUGAACGAUTT-3′	5′-AUCGUUCAGAUUCAUGGUGTT-3′
AMPKα	5′-GUGGCAGUUAAGAUCUUAATT-3′	5′-UUAAGAUCUUAACUGCCACTT-3′
Scrambled Control	5′-UUCUCCGAACGUGUCACGUTT-3′	5′-ACGUGACACGUUCGGAGAATT-3′

**Table 2:** Primer sequences for real-time PCR.

Gene	Forward Primer	Reverse Primer
LC3	5′-GATGTCCGACTTATTCGAGAGC-3′	5′-TTGAGCTGTAAGCGCCTTCTA-3′
Beclin1	5′-TTCAAGATCCTGGACCGAGTGAC-3′	5′-AGACACCATCCTGGCGAGTTTC-3′
ATG5	5′-TGAAGGAAGTTGTCTGGATAGCTCA-3′	5′-AAGGTCTGGTCCTTCCGCAGTC-3′
β-actin	5′-CCCATCTATGAGGGTTACGC-3′	5′-TTTAATGTCACGCACGATTTC-3′

## Abbreviations

RA	Rheumatoid Arthritis
ASIC1a	Acid-Sensing Ion Channel 1a
FoxO3a	Forkhead Box O 3a
ATG	Autophagy Related Genes
AMPK	AMP-Activated Protein Kinase
SiRNA	Small Interfering RNA
MDC	Monodansylcadaverine
TEM	Transmission Electron Microscopy
qRT-PCR	Quantitative Real-Time PCR
SD	Standard Deviation
